# Nutritionally Valuable Components and Heat-Induced Contaminants in Extruded Snack Products Enriched with Defatted Press Cakes

**DOI:** 10.3390/molecules29040791

**Published:** 2024-02-08

**Authors:** Antun Jozinović, Jelena Panak Balentić, Đurđica Ačkar, Mirta Benšić, Jurislav Babić, Veronika Barišić, Ante Lončarić, Borislav Miličević, Drago Šubarić

**Affiliations:** 1 Faculty of Food Technology Osijek, Josip Juraj Strossmayer University of Osijek, Franje Kuhača 18, 31000 Osijek, Croatia; ajozinovic@ptfos.hr (A.J.); jpanak@hotmail.com (J.P.B.); jbabic@ptfos.hr (J.B.); veronika.barisic@ptfos.hr (V.B.); ante.loncaric@ptfos.hr (A.L.); dsubaric@ptfos.hr (D.Š.); 2 School of Applied Mathematics and Informatics, Josip Juraj Strossmayer University of Osijek, Ljudevita Gaja 6, 31000 Osijek, Croatia; mirta@mathos.hr; 3 Faculty of Tourism and Rural Development Požega, Josip Juraj Strossmayer University of Osijek, Vukovarska 17, 34000 Požega, Croatia; bmilicevic@ftrr.hr

**Keywords:** snacks, oil press cakes, fibres, polyphenols, hydroxymethylfurfural, acrylamide

## Abstract

This research studies the influence of the addition of defatted press cakes (from the production of hazelnut, camelina, pumpkin, and hemp seed oil) on nutritionally important components: fibre, resistant starch, polyphenols, hydroxymethylfurfural (HMF), and acrylamide in directly and indirectly expanded snacks. The amounts of press cakes added to corn grits were 3, 6, and 9%. Extrusion was carried out in a laboratory single-screw extruder. For indirectly expanded products (SCFX), supercritical CO_2_ was injected during extrusion, and secondary expansion was completed in the microwave oven. The type and content of press cake, as well as the type of product, significantly influenced total polyphenol content and antioxidant activity. Press cakes increased the contents of both soluble and insoluble fibre (from 1.94% d. m. and 1.28% d. m. for extrudates without press cakes up to 3.17% d. m. and 6.94% d. m. for SCFX extrudates with press cakes, respectively), and resistant starch was not markedly influenced by their addition. The influence of the content of press cake on HMF was not significant, whereas the type of cake and the type of extrusion influenced HMF significantly. In a raw mixture of corn grits with 3% of pumpkin press cake, HMF was below the limit of detection, and the highest content was found in the classically extruded sample with the addition of 9% of camelina press cake (580 ppb). In all samples, the acrylamide content was below the limit of detection, indicating that safe products were obtained. This research shows potential for the implementation of supercritical CO_2_ extrusion in the production of safe, nutritionally improved snack products. Future research might bring about the design of cost-effective processes applicable in the industry.

## 1. Introduction

The modern Western societies are facing two major challenges: obesity and pollution of the environment. Modern lifestyle dictates the need for quickly and easily prepared meals, and highly processed foods—depleted of nutrients and high in energy—are commonly consumed among all age groups. The result is the epidemic of obesity and related diseases. In recent years, a lot of effort has been placed into the education of consumers, and awareness regarding nutritive value of food is slowly raising. However, there is still large demand for ready-to-eat and ready-to-heat food, but that which is nutritionally improved.

The environmental impact of food processing has been an issue for quite a long time. In recent years, many different approaches have been taken to resolve the problem of disposal of organic waste. All of them have had one common feature: organic material has been no longer considered a waste, but a by-product. Solutions have been found and applied, such as the use for animal feed or as an adsorbent in wastewater treatment and composting. However, the European Strategy 2020 [1] directs that these by-products should be primarily used as food and then as feed and for alternative purposes.

In the production of oil, press cakes remain after the extraction of oil. Generally, they are rich in protein, fibre, and bioactive compounds. For example, Sert et al. [2] reported that pumpkin seed press cake contains app. 60 g of protein/100 g, Gul et al. [3] reported app. 37% protein in hazelnut press cake, and Nakov et al. [4] reported 34 g protein/100 g of hemp press cake. Camelina press cake is rich in polyphenols, such as protocatechuic acid, catehin, and *p*-hydroxybenzoic acid, and contains glucosinolates [5]. There are indications that camelina reduces intestinal damage and might be used to treat persistent abdominal pain [6]. Furthermore, it has been reported that press cakes contain from 10% to up to 45% fibre, which was determined in hemp press cake [7]. This property is a good basis for the use of press cakes in processed foods to increase the intake of fibre by consumers in Western countries, where this nutrient is poorly consumed due to the extensive consumption of highly processed food. Although previously mentioned, authors made an attempt to use these by-products for food production or the extraction of protein, they are still predominantly used as feed for livestock [8].

Leonard et al. [9] reported that, for instance, the demand for use of hemp seed press cake for human nutrition is still very low, despite its high nutritional value (app. 30% protein, app. 46% fibre, fibre-bound polyphenols). Therefore, it is reasonable to incorporate by-products that are familiar to consumers into products, such as biscuits, muffins, snacks, etc. Snacks may be produced via extrusion.

Extrusion is a process in which materials are subjected to high temperatures, high pressures, and high shear. Although materials are exposed to these parameters for a short period of time, the process results in significant changes, such as plasticization, depolymerization, and complexation. Loss of heat-sensitive compounds may occur, although to a lesser extent than in other heat processes.

Leonard et al. [10] claim that extrusion distinguishes itself as the most important processing technology for the utilization of by-products, due to HTST (high temperature–short time) characteristics. Indeed, it has been successfully applied to produce extrudates with the addition of corn fibre [11], mango peel [12], cocoa husk [13], brewer’s spent grain, sugar beet pulp, apple pomace [14], etc. Fibre addition had a positive effect on antioxidant properties [11] and resistant starch content [13], but hardness of the extrudates increased [12], which may be resolved by the addition of pectin [14] or subsequent drying [12].

Depending on the parameters (pressure and temperature) and food composition, resistant starch may be formed during extrusion through the depolymerization of amylopectin, increase in amylose content, and formation of amylose–lipid complexes [15]; the digestibility of starch may increase due to its loss of crystalline structure as well as its gelatinization and depolymerization to oligosaccharides [16]. If proteins are present during extrusion, they compete with starch and fibre for water and undergo a series of structural changes, losing structure and fractionation. Amino acids released during this process may interact with starchy components, sugars and/or bioactive compounds. The reactions are complex and unpredictable, and the process may improve or impart the nutritional values of the products. Therefore, extrusion with supercritical CO_2_ was introduced by Rizvi and Mulaney in 1992. Due to the supportive plasticizing and expanding effect of supercritical CO_2_, it is possible to reduce the temperature of the process from the commonly applied temperature of 130–180 °C to approximately 60–80 °C, thus minimizing the degradation of heat-sensitive compounds [17].

In this regard, our team focused on the use of defatted press cakes from hazelnut, camelina, pumpkin, and hemp seed oil production during the making of extruded snacks. We applied the conventional process of direct expansion, and extrusion was supported with supercritical CO_2_ with secondary expansion by microwaves. Unlike previously cited research, we applied supercritical CO_2_ in a single-screw extruder, not using it as a direct plasticizing agent, but as an aid in the secondary expansion of the product, replacing additives that are normally used in such products. This opened a potential for the development of products that could be readily expanded by the consumer at home, using a simple and affordable process, allowing them to enjoy freshly prepared snack, with improved nutritional properties. We attempted to consider all important aspects: processing issues, sensory properties, degree of food enhancement, and safety of novel products.

In our previous study, we emphasized the importance of enriching products with nutritionally valuable substances and highlighted the advantages of extrusion and supercritical CO_2_ extrusion in the production of snacks [18]. In that paper, we focused on processing issues and sensory properties. This paper expands the above-mentioned topic and focuses on the nutritional value of snacks, with respect to dietary fibre, resistant starch, polyphenol content, and the safety of obtained snacks, i.e., HMF and acrylamide.

## 2. Results and Discussion

### 2.1. Dietary Fibre

**Figure 1:**
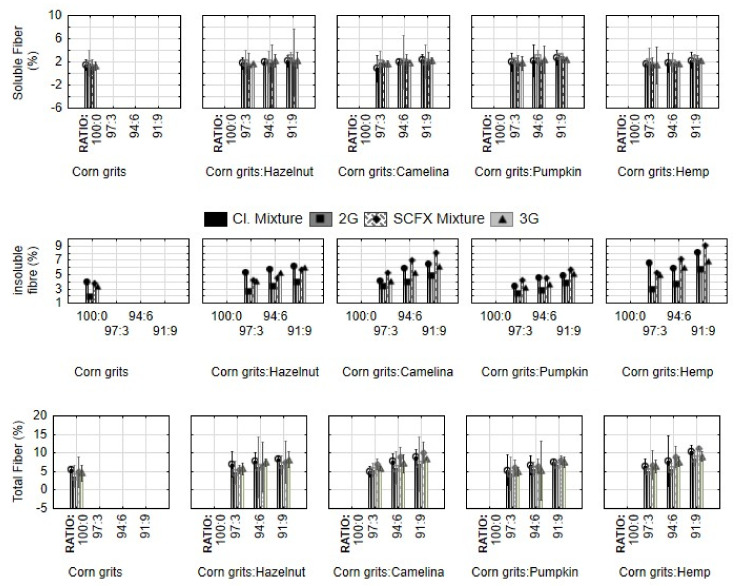
Fibre content in mixtures for extrusion and expanded snacks produced from corn grits with the addition of hazelnut, camelina, pumpkin, and hemp press cake in different proportions (3, 6, 9%). Cl. Mixture, mixtures of samples for 2G snacks; 2G, directly expanded snacks; SCFX Mixture, mixture for 3G snacks; 3G, indirectly expanded snack produced by supercritical CO_2_ extrusion and subsequent microwave expansion.

The results presented in Table 1 and Figure 1 show that press cakes are a valuable source of fibre. All four press cakes (hazelnut, camelina, pumpkin, and hemp) increased the contents of soluble fibre both in directly and indirectly expanded snacks, with the most pronounced effect of pumpkin cake. Soluble fibres have important functional properties, such as prebiotic effect, delayed gastric emptying, lower postprandial glucose levels, etc. [18]. Although extrusion has been used to increase the amount of soluble fibres at the expense of the insoluble fraction [19], the supercritical CO_2_ extrusion (SCFX) and microwave expansion in this research study did not have a marked effect on soluble fibre content. To transform insoluble fibres into soluble ones, they have to be partly hydrolysed and depolymerized in order to increase the porosity of the cell wall. To achieve this, high temperatures and pressures are needed during extrusion, and fibre-rich materials are often pre-treated with alkali and/or enzymes [19]. In our research, 2G products (directly expanded) indeed had lower contents of insoluble fibre than their 3G counterparts, due to more severe conditions of extrusion; however, the process did not influence their contents markedly.

Among the used by-products, the hemp cake had the most pronounced effect on the increase in insoluble fibre content. Our results are in accordance with the research of Tovar-Jimenez et al. [20], who reported a decrease in insoluble fibre content after extrusion and a microwave expansion of snacks with orange by-products, and Wang and Ryu [10], who reported that lower temperatures of extrusion do not induce the depolymerization of macromolecules and the conversion of insoluble to soluble fibre, as we observed for SCFX samples.

This research also supports the aforementioned richness of hemp press cake in fibre reported by Apostol et al. [7], since total fibre content was most significantly increased by the addition of hemp cake.

### 2.2. Resistant Starch

Among fibres, increased attention is being drawn to resistant starch. Although it is starch chemically, resistant starch is not digested until it reaches the colon, where it is fermented by intestinal microflora producing short-chain fatty acids and gases [21]. There is evidence that resistant starch decreases the insulin response and glycaemic index, and promotes the proliferation of intestinal microflora and mineral absorption [21]. This indicates that resistant starch could be used in functional foods.

**Figure 2:**
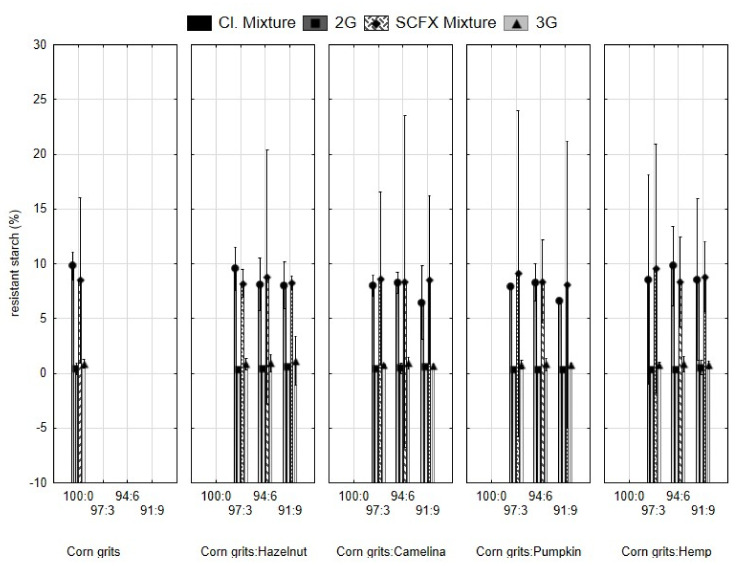
Resistant starch content in mixtures for extrusion and expanded snacks produced from corn grits with the addition of hazelnut, camelina, pumpkin, and hemp seed press cakes in different proportions (3, 6, 9%). Cl. Mixture, mixtures of samples for 2G snacks; 2G, directly expanded snacks; SCFX Mixture, mixture for 3G snacks; 3G, indirectly expanded snack produced by supercritical CO_2_ extrusion and subsequent microwave expansion.

Although it is significantly more resistant to heat than digestible starch, technologically, it behaves the same as digestible starch, which is important in flour-based products. Common fibre requires more water than normally used for such products, leading to difficulties with dough manipulation and reduced rising in bakery products. Resistant starch may be present in raw materials or can be formed during their processing, such as during extrusion. The content of resistant starch in food is influenced by non-starch components, and the interactions of starch with proteins, lipids, and fibres during processing may lead to changes in its content. All of these were reasons to monitor resistant starch content in this research study. The results are presented in Figure 2.

Resistant starch content was not significantly influenced by the type or the amount of added press cakes, unlike the extrusion, which reduced its content in all samples, with a more pronounced effect of direct expansion. During the process, the combined effects of high shear, high pressure, and high temperature cause gelatinization and depolymerization of starch molecules, making them more susceptible to enzyme attacks. Martinez et al. [22] and Arribas et al. [23] observed the same trend. The high temperature in the extruder barrel disrupts the crystalline structure of starch, breaks hydrogen bonds, and causes amylose to leach out. These processes result in a larger absorption of water into granules and swelling. The higher the temperature, the larger the extent of changes and starch transit from a crystalline to a fully amorphous state, which results in increased digestibility [10]. To obtain a higher content of resistant starch in extruded snacks, chemically modified starch (RS type 4) may be added before or during extrusion [24,25].

### 2.3. Total Phenolic Content and DPPH Radical Scavenging Activity

**Figure 3:**
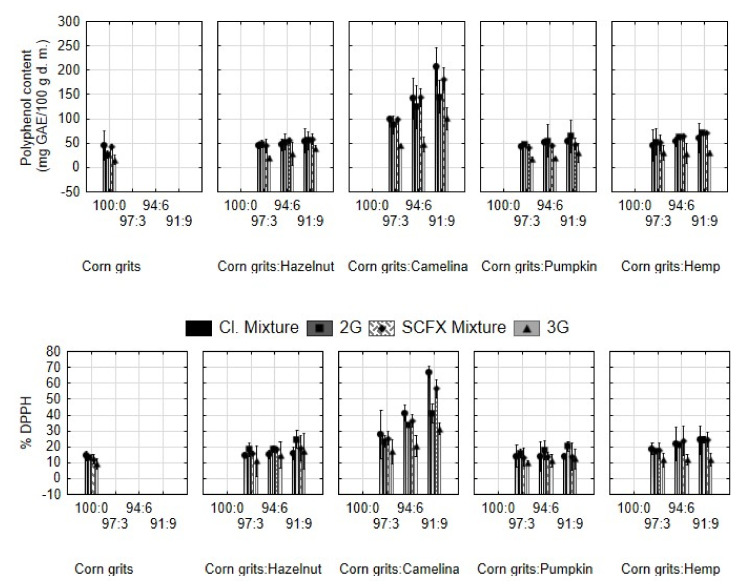
Polyphenol content and antioxidant activity (% DPPH) of mixtures for extrusion and expanded snacks produced from corn grits with the addition of hazelnut, camelina, pumpkin, and hemp seed press cakes in different proportions (3, 6, 9%). Cl. Mixture, mixtures of samples for 2G snacks; 2G, directly expanded snacks; SCFX Mixture, mixture for 3G snacks; 3G, indirectly expanded snack produced by supercritical CO_2_ extrusion and subsequent microwave expansion.

Along with fibre, press cakes are a valuable source of polyphenols [26,27]. Since these compounds have a beneficial effect on human health [28,29], this represents another aspect of the enhancement of the nutritional value of extruded snacks by incorporating press cakes. Results for total polyphenol content (TPC) and antioxidant activity (AO) are shown in Figure 3. TPC and AO increased with the addition of defatted cakes. The highest values were observed for samples with camelina cake, where TPC and AO increased proportionally with the addition of the by-product.

2G extrusion further increased TPC and AO values in samples with the addition of hazelnut, pumpkin, and hemp cake; however, with camelina cake, these values decreased in the extruded samples compared to mixtures. The increased values of TPC and AO after extrusion were observed in other research studies as well [30,31,32]. These authors explained that chemical reactions in food inside the extruder are unpredictable and cannot be controlled or influenced, as every food compound acts differently when temperature and pressure are increased. Our previous research with other raw materials showed that TPC and AO decreased after the extrusion process [33]. The observed increase in the TPC value after extrusion may be due to the release of bound polyphenols from a cell wall membrane [10], although Folin’s reagent may have interacted with proteins as well [9], thus only seemingly increasing the TPC. The increase in AO may be a result of the combined activity of liberated polyphenol and bioactive compounds and pigments formed due to Maillard reactions [10].

On the other hand, the decreased content of TPC in extruded samples with camelina cake could be caused by the breakage of covalent bonds, decomposition of heat-sensitive compounds, and decreased stability of the copigment complexes, as well as decarboxylation, de-esterification, and Maillard reactions [34].

Microwaves decreased the polyphenol content and antioxidant activity in 3G snacks compared to 2G, which is consistent with the research of Navarro-Cortez et al. [35]. Microwave heating is based on internal heat generation through ionic conduction, dipole polarization, and intermolecular friction, where the shape, size, and dielectric properties of a material determine the way it is heated. During the microwave heating of extruded pellets, water molecules vibrate and evaporate, thus inducing expansion, and heat degradation of polyphenols occurs. All three grouping variables influenced the measured values significantly (*p* < 0.05) (Table 1). Also, the coefficient of correlation showed statistical significance between TPC and AO, which is consistent with the above-mentioned research.

### 2.4. Acrylamide and Hydroxymethylfurfural

Press cakes are not just sources of bioactive compounds. They contain high amounts of proteins as well. This makes them possible triggers for browning reactions in which acrylamide and hydroxymethylfurfural are formed. Acrylamide is carcinogenic and the dietary exposure to it is of major concern [36]. Therefore, its content in different types of products, from coffee to French fries and breakfast cereals, is regulated by the EU regulation 2017/2158 [37]. Acrylamide forms during thermal processing (such as baking, cooking, frying) of foods that contain proteins and carbohydrates. Amino acids enter the Maillard reaction with reducing sugars to form a Shiff base, which undergoes cyclization and acid-catalysed rearrangement that produces Amadori and Heyns products. These compounds undergo different reactions (deamination, dehydration, enolization, fragmentation), producing carbonyl compounds that may undergo further reactions with free amino acids (predominantly asparagine) and produce acrylamide [38]. Since we introduced proteins by adding oil press cakes to the corn grits, the contents of acrylamide and hydroxymethylfurfural were studied in this paper as well. Results for acrylamide are not presented because all values were under the limit of detection (<3.79 µg/kg), indicating that the extrusion processes with these raw materials can provide products that are safe for the consumer (minimum LOD according to EU 2017/2158 is 6 µg/kg [37]). The assumption is that there is not enough precursor in the raw material that causes the formation of AA both with classical extrusion and SCFX process with the subsequent expansion via microwaves.

**Figure 4:**
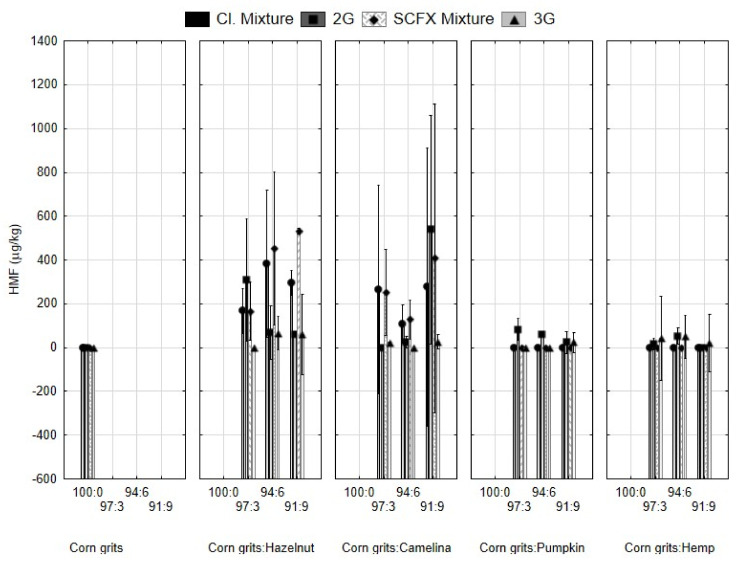
Hydroxymethylfurfural (HMF) content in mixtures for extrusion and expanded snacks produced from corn grits with the addition of hazelnut, camelina, pumpkin, and hemp seed press cakes in different proportions (3, 6, 9%). Cl. Mixture, mixtures of samples for 2G snacks; 2G, directly expanded snacks; SCFX Mixture, mixture for 3G snacks; 3G, indirectly expanded snack produced by supercritical CO_2_ extrusion and subsequent microwave expansion.

Results for HMF content are presented in Figure 4. It is evident that HMF was below the LOD in the control sample of corn grits. HMF was detected and quantified in mixtures with hazelnut and camelina cake, but not with the addition of pumpkin and hemp cake. This indicates that HMF was formed in raw materials, namely hazelnuts and camelina. The reason for this may be due the temperature-induced changes that occurred in the starting material (seeds and nuts) during the drying and storing of the raw material and due to oil production. Even though cold pressing was applied during oil extraction, the temperature of the cake elevated to values between 40 and 50 °C.

The increase in the HMF content was not proportional to the addition of press cakes. This could be due to the inhomogeneity of the mixtures (indicated by the standard deviation on the graph). In samples with the addition of pumpkin and hemp, there was a slight increase in HMF after extrusion. However, the values determined in 3G products were slightly lower than in the 2G snacks.

**Figure 5:**
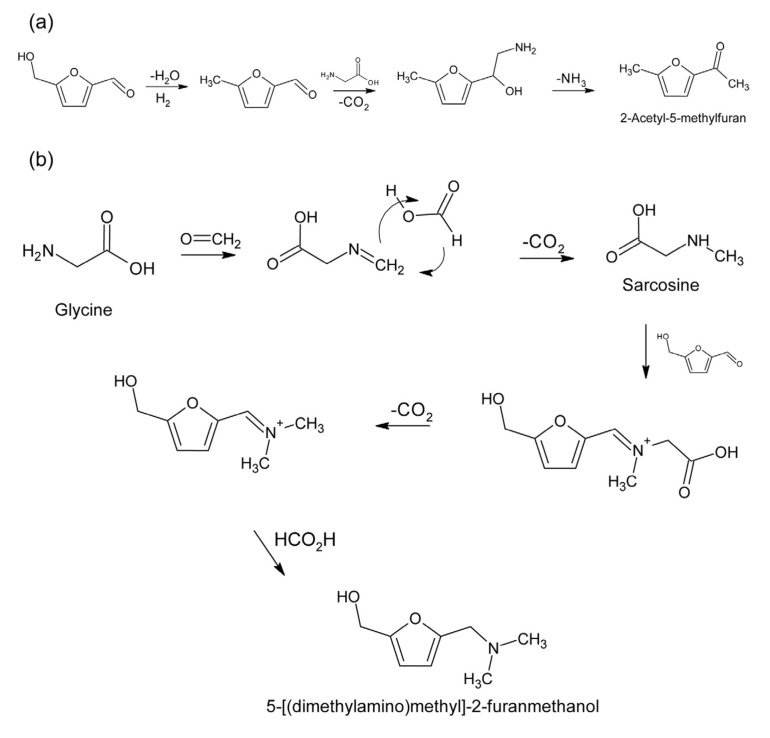
Thermal decomposition of HMF in presence of glycine; (**a**) formation of 2-acetyl-5-methylfuran, (**b**) formation of 5-[(dimethylamino)methyl]-2-furanmethanol.

It is known that protein-rich raw materials are good precursors for HMF formation by processing at high temperatures, but, in spite of this, the values were so low that it could be said to be negligible. In the samples with hazelnut and camelina cake, the HMF content decreased after the extrusion process. It is assumed that there was a thermal decomposition of HMF in the presence of glycine to 2-acetyl-5-methylfuran and 5-[(dimethylamino) methyl]-2-furanmethanol (Figure 5) [39] because hazelnut and camelina contain glycine [40]. Statistical analysis showed that the type of cake and type of extrusion had a statistically significant effect on HMF (*p* < 0.05) (Table 1).

## 3. Materials and Methods

### 3.1. Materials

Materials used in this study were corn grits, and hazelnut, camelina, pumpkin, and hemp seed press cakes. Press cakes were obtained by screw pressing in a screw expeller (Model SPU 20; Senta, Serbia) and were defatted via supercritical CO_2_ extraction (custom-made extractor described by Jokić et al. [41]). These processes are described in detail in our previous article, with the same preparation for usage in the extrusion process, along with the approximate composition [18].

Gallic acid, Folin–Ciocalteu, DPPH, high-purity acrylamide, ^13^C_3_-acrylamide, and 5-hydroxymethylfurfural were purchased from Sigma-Aldrich (Diesenhofen, Germany). HPLC gradient grade acetonitrile and methanol were purchased from J.T. Baker (Deventer, The Netherlands).

### 3.2. Sample Preparation

Obtained by-products were added to corn grits in 0, 3, 6, and 9%. The moisture of the samples was adjusted to 15% (marked as Cl. Mixture, later used for production of 2G—directly expanded snacks) and 25% (SCFX Mixture, later used for 3G—indirectly expanded snacks). To enable expansion, mixtures for direct expansion (2G) were prepared with the addition of pectin. A more detailed description of the sample preparation and the approximate composition of the raw materials can be found in our previous article [18].

### 3.3. Extrusion

2G products were produced using Cl. Mixture. Extrusion was performed in a laboratory single-screw extruder (Brabender GmbH, Model 19/20DN, Duisburg, Germany), at a temperature profile of 135/170/170 °C, with screw 4:1, and 4 mm die [14].

3G products were produced using SCFX mixture in a laboratory single-screw extruder (Brabender GmbH, Model 19/20DN, Duisburg, Germany) modified for supercritical CO_2_ extrusion. The extrusion parameters to produce 3G products were 90/110/120 °C; screw 3:1; 4 mm die; air pressure: 2.2 bar; CO_2_ pressure: 140 bar; and CO_2_ heating bath: 90 °C [18]. Supercritical CO_2_ was used as a blowing agent, and no additives or any other chemical substances were added.

### 3.4. Dietary Fibre

Dietary fibre (total, soluble, and insoluble fibre) was determined via the AOAC 991.43 method [42], using the Megazyme kit (Bray, Ireland). Briefly, in the first step, starch is depolymerized by cooking with heat-stable α-amylase at 100 °C. Afterwards, proteins are removed by incubation at 60 °C with protease, and starch fragments are further hydrolysed to glucose with amyloglucosidase. The residue is filtered, washed with ethanol and acetone, and dried. One parallel is used to determine protein (Kjedahl method) and the other to determine ash content (525 °C, 5 h). Insoluble fibre is the weight of the filtered and dried residue minus the weight of the protein and ash.

Soluble fibre is determined from the filtrate after precipitation with ethanol, filtration, washing, and drying with the same principle used for insoluble fibre.

### 3.5. Resistant Starch

Resistant starch (RS) content was determined according to the AOAC 2002.02 method [43]. Briefly, non-resistant starch was hydrolysed to glucose via incubation with pancreatic α-amylase and amyloglucosidase (AMG) for 16 h at 37 °C. Resistant starch was recovered as a pellet after washing with ethanol and centrifugation, dissolved in KOH, and hydrolysed to glucose with AMG. D-glucose was measured spectrophotometrically after the reaction with the glucose oxidase/peroxidase reagent (GOPOD), and this was a measure of the RS content.

### 3.6. Total Phenolic Content and DPPH Radical Scavenging Activity

The total phenolic content (TPC) was determined according to the Folin–Ciocalteu colorimetric method, with gallic acid (GA) as a standard. The samples (1 g) were extracted with methanol/water 80:20 (10 mL) for 2 h at an ambient temperature and were filtered (Whatman Nr 1, Whatman International, Maidstone, UK). A 300 µL extract was added to 1.5 mL diluted Folin–Ciocalteu reagent, with 1.5 mL of sodium carbonate being added after 5 min; after 90 min of incubation, absorbance was read at 725 nm with 80% methanol as a reference, and the results were expressed as mg gallic acid equivalents (GAE)/100 g d. m.

DPPH radical scavenging activity of sample solutions was estimated using the stable radical DPPH. The methanol DPPH solution (3.9 mL) was added to the extract (200 µL), shaken vigorously, and allowed to stand in the dark for 30 min. The control sample was prepared using 80% methanol instead of the sample. The absorbance of the reaction mixtures was read at 517 nm, and scavenging activity was calculated according to Equation (1), as follows:

(1) $$scavenging\;activity\;\left(\%\right)=\frac{A517\;\left(control\right)-A517\;(sample)}{A517\;(control)}\times 100$$

Both methods are described in detail by Wang and Ryu [10].

### 3.7. Contents of Acrylamide and Hydroxymethylfurfural

Acrylamide and hydroxymethylfurfural contents were determined simultaneously according to the method developed in our laboratory [44]. After methanolic extraction and purification, samples were reconstituted in water/acetonitrile (50:50 *v*/*v*), filtered through a 0.45 μm PVDF filter, and analysed in an HPLC system (PerkinElmer, Walthman, MA, USA) consisting of a binary pump, an autosampler and column oven (temperature controlled), and that was coupled to an API 2000 MS/MS (Sciex, Framingham, MA, USA) detector. Ionization was performed with an APCI (atmospheric pressure chemical ionization) interface, and the separation of ions was carried out with triple quadrupole. Column Zorbax Extend-C18 (250 × 4.6 mm, 5 µm) (Agilent, Santa Clara, CA, USA) was used for analytical separation.

All analyses were carried out in five replicates.

### 3.8. Statistical Analysis

The software programme STATISTICA 13.3 (StatSoft, Inc., Tulsa, OK, USA) was used for statistical analysis. The experimental data were analysed using analysis of variance (one-way ANOVA) with corrections carried out with Welch’s F test. The results are presented using descriptive statistics and the method of categorized means (interaction) plots, with a significance of *p* < 0.05.

## 4. Conclusions

Press cakes explored in this research comprised hemp seeds, camelina, hazelnut, and pumpkin seeds, which were successfully applied as agents for the nutritional improvement of directly (2G) and indirectly expanded snacks (3G), with no adverse effects on their safety. The addition of press cakes resulted in increased dietary fibre and polyphenol content as well as an increased antioxidant activity of 2G and 3G snacks. All obtained products may carry a nutrition claim “source of fibre” according to Regulation (EC) No 1924/2006 [45] and Regulation (EU) No 1047/2012 [46], since fibre content in all samples exceeded 3 g/100 g. Regardless of the samples and applied processes, the contents of AA were below the values regulated by the EU in all analysed samples, and HMF content was not high, showing no potential health risk for consumers. Selected press cakes are a good source of polyphenols and dietary fibre that may be used for the enrichment of extruded snack products, facilitating environmentally friendly and efficient disposal of by-products of the oil industry.

If sensory properties are considered (shown in our previous article: Panak Balentić et al.) along with the nutritional improvement of snack products, six samples can be distinguished with the potential for industrial applications: corn/hazelnut 94:6; corn/camelina 97:3 and 94:6; corn/pumpkin 97:3 and 94:6; and corn/hemp 91:9.

However, there are still limitations to the practical applications of this research study in the industry. Supercritical CO_2_ technology is still costly for the industrial implementation of this method, and further research should seek to explore potential solutions for cost-effective applications of this environmentally friendly process in the food industry.

## Figures and Tables

**Table 1:** One-way ANOVA correction with the Welch’s F test.

Variables	Grouping Variables	Welch *p* *
Polyphenol content (mg GAE/100 g d. m.)	Type of cake	**<0.000001**
	Content of cake	**0.000115**
	Type of product	**0.000009**
% DPPH	Type of cake	**<0.000001**
	Content of cake	**<0.000001**
	Type of product	**0.000203**
HMF (μg/kg)	Type of cake	**0.000004**
	Content of cake	0.358336
	Type of product	**0.000195**

* Values in bold indicate statistical significance at *p* < 0.05.

## Data Availability

The data presented in this study are contained within this article.
